# Diabetes severity measured by treatment control status and number of anti-diabetic drugs affects presenteeism among workers with type 2 diabetes

**DOI:** 10.1186/s12889-021-11913-3

**Published:** 2021-10-16

**Authors:** Takahiro Mori, Tomohisa Nagata, Masako Nagata, Kenji Fujimoto, Yoshihisa Fujino, Koji Mori

**Affiliations:** 1 Department of Occupational Health Practice and Management, Institute of Industrial Ecological Sciences, University of Occupational and Environmental Health, Japan, 1-1 Iseigaoka Yahatanishi-ku, Kitakyushu, Fukuoka, Japan; 2 Data Science Center for Occupational Health, University of Occupational and Environmental Health, Japan, 1-1 Iseigaoka Yahatanishi-ku, Kitakyushu, Fukuoka, Japan; 3 Department of Environmental Epidemiology, Institute of Industrial Ecological Sciences, University of Occupational and Environmental Health, Japan, 1-1 Iseigaoka Yahatanishi-ku, Kitakyushu, Fukuoka, Japan

**Keywords:** Presenteeism, Severity, Treatment control, Anti-diabetic drug, Combination therapy

## Abstract

**Background:**

The number of people with diabetes is increasing and resulting in major economic losses. Presenteeism accounts for the majority of economic losses, so measures against presenteeism are important. This study investigated the relationship between severity of type 2 diabetes and presenteeism.

**Methods:**

A cross-sectional study was conducted among workers over 40 years of age. Participants were classified as normal group or diabetic treatment group using their medical examination results and health insurance claims data. Diabetic treatment groups were described by degree of treatment control: Good (HbA1c < 7%), Intermediate (7% ≤ HbA1c < 8%), and Poor (8% ≤ HbA1c). Therapy type was also divided into monotherapy and combination therapy. Logistic regression analysis was performed to predict presenteeism loss using the Quantity and Quality method.

**Results:**

Data on 13,271 workers were analyzed. Presenteeism loss was significantly higher in all treatment control groups compared with the normal group, particularly for the intermediate and poor control groups. The monotherapy group did not differ from the normal group, but presenteeism loss was significantly higher in the combination therapy group than the normal group.

**Conclusions:**

Presenteeism loss in workers with diabetes may be affected by diabetes severity, and even if treatment control were good, presenteeism loss could occur when the number of anti-diabetic drugs was high. Therefore, it is important to provide early intervention and continuous support as a preventive measure against not only diabetes and diabetes-related complications but also presenteeism.

**Supplementary Information:**

The online version contains supplementary material available at 10.1186/s12889-021-11913-3.

## Background

The number of people with diabetes is increasing. According to global reports of the International Diabetes Federation (IDF), 463 million people were diagnosed with diabetes in 2019. It is estimated that the number will increase to 578 million by 2030 and 629 million by 2045 if effective measures are not taken [1].

The increasing number of people diagnosed with diabetes constitutes a significant economic loss for employees, employers, and society. The American Diabetes Association has estimated the economic cost of diabetes in the United States every five years since 1997 [2–6], and the latest (2017) cost was $ 327 billion [6]. Similar estimates have also been made in European countries [7–11]. In Japan, it have been reported that estimates of diabetes burden including indirect costs for the period 2010–2030 measured in real USD with the base year 2010 have been 667 billion USD, which was higher than other chronic conditions such as ischemic heart disease, cerebrovascular disease, chronic obstructive pulmonary disease, breast cancer [12]. However, most studies have estimated losses according to the number of only those diagnosed with diabetes, and they have not included undiagnosed patients. The IDF reports that almost half of all adults with diabetes worldwide have not been diagnosed, and therefore including those individuals would further increase the losses [13].

Economic costs of illness consist of direct and indirect costs. Absenteeism and presenteeism, which are included in indirect costs, are often evaluated as productivity losses due to workers’ health problems. Absenteeism refers to “absence from work due to health problems,” while presenteeism is defined as “health-related productivity loss while at paid work” [14].

According to a survey of employees of large US companies, presenteeism due to diabetes accounted for 62% of the total costs and 87% of the indirect costs of diabetes [15]. Therefore, as a measure of diabetes in society as a whole—and in companies—it is important not only to reduce medical costs but also to take measures against presenteeism, which accounts for the majority of all health-related economic losses. Particularly, it is expected that the number of individuals working while being treated for diabetes will increase due to social changes such as the extension of retirement age because of the declining birthrate and aging population in Japan. Prevention of chronic diseases—including diabetes—and measures against presenteeism are becoming more and more important in the workplace.

Many studies have pointed out that diabetes causes presenteeism, but there were some problems in that undiagnosed diabetes was not included, and treatment status analysis was not always performed. Therefore, as a previous study of this research, we defined the classification of diabetes for Japanese workers according to the results of medical examinations and using health insurance claims data, and we analyzed the relationship between diabetes status and presenteeism. We reported that presenteeism occurred significantly in the diabetic drug treatment group compared with that in the normal group. However, no presenteeism occurred in the borderline group and in the untreated diabetic group including undiagnosed patients [16]. It is suggested that presenteeism does not occur just by having diabetes since diabetes is basically asymptomatic in the early stages, and the cause of presenteeism in the diabetes treatment group could be that they already had symptoms or had complications related to the influence of the severity of diabetes and the influence of the diabetic drug itself.

As a background to the occurrence of presenteeism due to diabetes, the existence of diabetes-related complications and complications has been reported. Previous studies have investigated the direct causes such as hypoglycemia [17–21], diabetic neuropathy [22–25], diabetic foot ulcer [26], diabetes-associated stress [27, 28], complications of mood disorders such as depression [29, 30], and the number of tolerability issues with anti-diabetic drugs [31]. With regard to hypoglycemia, it has been reported that even if the symptoms are mild or if hypoglycemia occurs at night, the effects on work the next day are not small. Regarding the diabetic neuropathy, it has been reported that the intensity of pain is related to diabetic neuropathy, and that the stronger the pain, the higher presenteeism occurs. Similarly, diabetes patients with diabetic foot ulcers have higher presenteeism than the patients without diabetes or the diabetes patients without foot ulcers. People with diabetes are more likely to have mood disorders such as depression and stress, and it has been found that people with both diabetes and mood disorders have higher presenteeism than those with either diabetes or mood disorder only. As the number of tolerability issues with anti-diabetic drugs such as hypoglycemia, headache, water retention (edema), and weight gain, etc., increased, presenteeism worsened.

However, few studies have investigated the relationship with severity. Here, the severity of diabetes means two of “treatment control status” and “number of anti-diabetic drugs”. Because the severity of diabetes is evaluated by “treatment control status” according to test values such as fasting blood glucose and hemoglobin A1c (HbA1c). But as a general treatment flow, in non-insulin dependent states such as type 2 diabetes mellitus, if exercise or diet does not improve glycemic control, monotherapy is started. Moreover, if treatment control is not possible with monotherapy, patients need to be treated with two or more drugs [32], and the severity is also evaluated in terms of the number of anti-diabetic drugs. Thus, it is considered appropriate to express the severity of diabetes in terms of treatment control status according to test values and number of anti-diabetic drugs. It is difficult to distinguish between the influence of the severity and the influence of the drug itself.

The purpose of this study was to investigate the relationship between two severities—the treatment control status and the number of anti-diabetic drugs—and presenteeism for type 2 diabetes mellitus.

## Methods

A cross-sectional study was conducted of workers aged 40 or over from 7 private companies in various industries such as electrical appliances, pharmaceuticals, the wholesale industry, and the food industry in Japan. The data were obtained from the results of annual medical examinations in each company in 2016, questionnaire surveys conducted with workers, and health insurance claims. According to Article 44 [1] of the Occupational Safety and Health Regulations, blood tests are a legal requirement for those aged 40 or over in medical examinations. In addition, the prevalence of diabetes increases with age, and prevalence under 40 years is approximately one-third that of those in their 40s [33]. For these reasons, we considered that including workers under 40 would affect the analysis, and therefore we limited our focus to those aged 40 or over in this study. In conducting this study, we explained the purpose to the management and workers via e-mail, intranet homepage, or the Safety and Health Committee, and the consent of the workers was obtained.

**Fig. 1:**
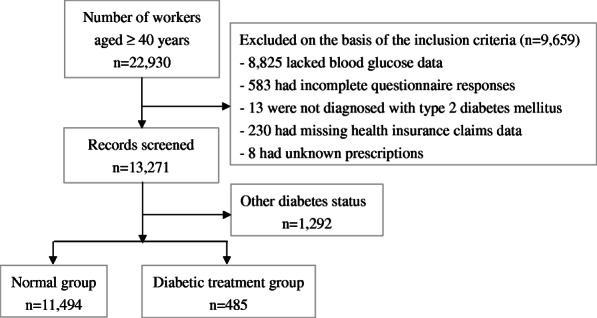
Participant classification flowchart

A total of 22,930 workers aged 40 years or older were selected. We excluded 8825 participants with data for only one test and those with casual blood glucose instead of fasting blood glucose because both fasting blood glucose and HbA1c were used in this study. In addition, we excluded 583 who had deficiencies in the questionnaire survey, 13 who were diagnosed with type 1 diabetes or other specified diabetes because this study targeted type 2 diabetes mellites, 230 whose treatment status was unknown and 8 patients receiving treatment had unknown prescriptions. We then analyzed 13,271 participants (Fig. 1).

The research protocol was approved by the Ethics Committee of Medical Research, University of Occupational and Environmental Health, Kitakyushu, Japan (H26–026).

### Classification of diabetes status

We categorized diabetes status of participants with the results of annual medical examinations and the health insurance based on the diabetes diagnosis criteria of the Japan Diabetes Society [34]. The participants with fasting blood glucose of less than 110 mg/dL and HbA1c [National Glycohemoglobin Standardization Program (NGSP)] (hereinafter HbA1c) of less than 6.0% were defined as the normal group. From the health insurance claims, if participants had taken anti-diabetic drugs from 3 months before the questionnaire through to the response month, they were classified as being in a diabetic treatment group regardless of their blood tests. Participants with insulin treatment were also included in the diabetic treatment group. In addition, information on the diagnosis name and prescription content was also collected from the health insurance claims. The reason for confirming the prescription history for 3 months was that the prescription of anti-diabetic drugs is mostly for 90 days or less in Japan [35]. Regarding insulin treatment, some previous studies reported that it was a factor in the occurrence of presenteeism [28, 36], but no significant difference was found in the insulin treatment group in our previous studies [16]. Thus we considered that insulin had almost no effect and decided to include it in the diabetic treatment group in this study. We did not consider typical symptoms of diabetes such as dry mouth or polyuria.

### Classification of diabetes severity

We classified the diabetic treatment group as follows for two severities: the treatment control and the number of anti-diabetic drugs. Regarding the treatment control, treatment control targets are the same in the United States [32] and Japan [34] where the target for prevention of complications is HbA1c < 7%, and if it is difficult to strengthen the treatment due to side effects such as hypoglycemia, the target is HbA1c < 8%. Therefore, we divided participants into three groups according to treatment control targets: good control group—HbA1c < 7%; intermediate control group—7% ≤ HbA1c < 8%; and poor control group—8% ≤ HbA1c. Next, regarding the number of anti-diabetic drugs, we classified as monotherapy group or combination therapy group if the participants were taking two or more drugs. Currently, some compounding drugs for diabetes are used, and participants taking these compounding drugs were included in the combination therapy group as two types of drugs were essentially prescribed.

### Assessment of Presenteeism

We defined productivity loss due to presenteeism as “presenteeism loss” and evaluated the loss using the Quantity and Quality (QQ) method [37]. The evaluation using this method was performed through the following steps. First, we asked whether participants had any health problems or conditions during their work in the past month. If the answer was “no,” the presenteeism loss was set to zero. If the answer was “yes,” we asked the participants to identify their health problems from a list of 14 conditions and to select the one condition that most affected their work. If the conditions did not affect their work, the presenteeism loss was also set to 0. The 14 conditions were as follows: (2) troubled by allergies (e.g. hay fever); [1] skin diseases/itchiness (e.g. eczema, atopic dermatitis); [3] disorders caused by infections (e.g. cold, influenza, gastroenteritis); [4] gastrointestinal disorders (e.g. recurrent diarrhea, constipation); [5] pain in arm and leg joints or lack of mobility (e.g. arthritis); [6] back pain; [7] painful neck or stiff shoulder; [8] headaches (e.g. migraine, chronic headache); [9] tooth trouble (e.g. toothache); [10] mental health problems (e.g. depression, anxiety); [11] insomnia, insufficient sleep; [12] a sense of weariness or fatigue; [13] eye problems (e.g. loss of vision, eyestrain, dry eye, glaucoma); and [14] other.

Second, we asked participants to describe the quantity and quality of the work when they had the identified problem compared with those when they had no problems. The answers were scored from 0 (unable to work at all) to 10 (normal). Finally, presenteeism loss was calculated using the following equation:

Presenteeism loss = 100 – Quantity (range: 0–10) × Quality (range: 0–10).

In our previous study [16], the top 10% with a presenteeism loss score was defined as high presenteeism loss, and the top 20% was defined as moderate presenteeism loss, but similar results were obtained with these two indicators. Therefore, we set moderate presenteeism loss, a score of 36 or higher, which was the top 20%, as the outcome in this study.

### Statistical analysis

Participant characteristics were summarized using means and standard deviations (SDs) for continuous variables and percentages for categorical variables. The presenteeism loss was calculated in each treatment control group compared with that of the normal group, and was also calculated for each therapy type compared with that of the normal group. Furthermore, the presenteeism loss in each treatment control group of combination therapy was also calculated. We performed logistic regression analysis with each treatment control group, number of anti-diabetic drugs, and each treatment control group of combination therapy as the independent variable, and presenteeism loss using the QQ method, that is, 36 or higher as the dependent variable. For all analyses, the normal group was the reference category, and age, sex, employment status, occupation, and company were adjusted. The odds ratios (OR) of the crude model and adjusted ORs and corresponding 95% confidence intervals (CIs) were calculated. In all analysis, *p*-values < 0.05 were considered statistically significant. All analyses were performed using STATA Version 16 (StataCorp LLC, College Station, TX).

Sensitivity analysis were performed by the same analysis for high presenteeism loss of the top 10% with a presenteeism loss score of 51 or higher in order to confirm whether the same result could be obtained even if the outcome was changed.

## Results

11,494 were classified in the normal group and 485 in the diabetic treatment group (Fig. 1). Table 1 shows the characteristics of the participants. Among the diabetic treatment groups, 300 were in the “good control” group, 105 in the “intermediate control” group, and 80 in the “poor control” group. In addition, 190 participants were receiving monotherapy, and 146 of them were in the good control group. There were 295 individuals in the combination therapy group (including those taking compounding drugs), and the proportion of combination therapy increased in the intermediate and poor control groups.

**Table 1:** Baseline characteristics of the participants

	Normal group *n* = 11,494	Good control group *n* = 300	Intermediate control group *n* = 105	Poor control group *n* = 80
Age, years, mean (SD)	49.0 (5.7)	53.3 (5.2)	53.2 (4.9)	51.3 (5.1)
Gender, %				
Male	76.9	95.7	92.4	93.7
Female	23.1	4.3	7.6	6.3
Employment status, %				
Full-time	97.8	96.9	98.0	98.7
Contract	2.0	2.8	2.0	1.3
Part-time or Temporary	0.1	0.3	0	0
Occupation, %				
Managerial	39.7	42.1	41.3	26.9
Clerical	15.9	10.2	10.9	11.9
Sales	20.5	34.6	26.1	46.3
Research & Development	10.5	4.3	7.6	3.0
Engineering	4.6	3.1	3.3	1.5
Production line	8.1	4.7	8.7	10.4
Other	0.8	0.8	2.2	0
Smoking status, %	20.1	31.7	31.4	28.8
BMI (kg/m^2^), mean (SD)	23.0 (3.1)	26.5 (4.3)	26.8 (3.8)	28.1 (3.9)
Fasting blood glucose (mg/dL), mean (SD)	91.6 (7.6)	118.9 (20.3)	142.1 (25.2)	184.0 (46.9)
HbA1c (NGSP) (%), mean (SD)	5.4 (0.3)	6.3 (0.4)	7.4 (0.3)	9.2 (1.4)
Therapy type, %				
Monotherapy	–	48.7	29.5	16.3
Combination therapy	–	51.3	70.5	83.8
Quantity of work, mean (SD)	9.0 (1.7)	8.9 (1.7)	8.7 (2.1)	8.7 (2.2)
Quality of work, mean (SD)	8.9 (1.8)	8.9 (1.8)	8.6 (2.1)	8.4 (2.4)
Presenteeism loss, mean (SD)	16.8 (24.4)	17.6 (24.8)	21.8 (27.7)	22.7 (30.7)
Symptom days in the past month, mean (SD)	8.3 (10.4)	9.1 (10.9)	9.3 (11.0)	9.0 (10.2)

SD, standard deviation; BMI, body mass index; HbA1c, hemoglobin A1c,

NGSP, National Glycohemoglobin Standardization Program; Combination therapy, 2 or more anti-diabetic drugs; Presenteeism loss, productivity loss due to presenteeism

Presenteeism loss = 100 − Quantity (range: 0–10) × Quality (range: 0–10)

### Occurrence of presenteeism loss for each treatment control status

In the adjusted model, the odds of presenteeism loss were significantly higher in all control groups, the good control group (OR 1.48, 95%CI 1.11–1.96, *p* = 0.007), the intermediate control group (OR 1.92, 95%CI 1.24–2.98, *p* = 0.003), and the poor control group (OR 1.80, 95%CI 1.08–3.02, *p* = 0.024) than in the normal group. In particular, the odds were higher in the intermediate and poor control groups (Table 2).

**Table 2:** Relationship between each treatment control and presenteeism loss

	n	mean (SD) of presenteeism loss	%	Crude model			Adjusted model		
				OR	95% CI	*p*-value	OR	95% CI	*p*-value
Normal group	11,494	16.8 (24.4)	24.2	reference			reference		
Good control group	300	17.6 (24.8)	27.3	1.18	0.91–1.52	0.213	1.48	1.11–1.96	0.008
Intermediate control group	105	21.8 (27.7)	34.3	1.63	1.24–2.45	0.018	1.92	1.24–2.98	0.003
Poor control group	80	22.7 (30.7)	33.8	1.60	1.00–2.54	0.049	1.80	1.08–3.02	0.024

Adjusted model controlled for sex, age, employment status, occupation and company

Presenteeism loss, productivity loss due to presenteeism; SD, standard deviation; OR, odds ratio; CI, confidence interval

Sensitivity analysis were performed with high presenteeism loss, the top 10% with a presenteeism loss score of 51 or higher showed no significant difference, but the odds ratio tended to increase as the treatment control worsened (Supplemental Table 1).

### Occurrence of presenteeism loss for number of anti-diabetic drugs

In the adjusted model, there was no significant difference between the monotherapy and the normal group, but the odds of presenteeism loss were significantly higher in combination therapy (OR 1.82, 95%CI 1.38–2.39, *p* < 0.001). Regarding each treatment control group of combination therapy, the odds of presenteeism were significantly higher in all groups, the good control group (OR 1.55, 95%CI 1.05–2.29, *p* = 0.027), the intermediate control group (OR 2.12, 95%CI 1.27–3.55, *p* = 0.004), and the poor control group (OR 2.09, 95%CI 1.22–3.58, *p* = 0.007). In particular, the odds were higher in the intermediate and poor control groups (Table 3).

**Table 3:** Relationship between the number of anti-diabetics and presenteeism loss

	n	mean (SD) of presenteeism loss	%	Crude model			Adjusted model		
				OR	95% CI	*p*-value	OR	95% CI	*p*-value
Normal group	11,494	16.8 (24.4)	24.2	reference			reference		
Monotherapy group	190	17.1 (24.1)	26.8	1.15	0.83–1.59	0.400	1.35	0.95–1.92	0.098
Combination therapy group	295	20.8 (27.9)	31.9	1.46	1.14–1.88	0.003	1.82	1.38–2.39	< 0.001
Good control group	154	18.3 (25.6)	27.9	1.21	0.85–1.73	0.286	1.55	1.05–2.29	0.027
Intermediate control group	74	23.9 (29.0)	36.5	1.80	1.12–2.89	0.015	2.12	1.27–3.55	0.004
Poor control group	67	23.3 (31.3)	35.8	1.75	1.06–2.89	0.029	2.09	1.22–3.58	0.007

Adjusted model controlled for sex, age, employment status, occupation and company

Presenteeism loss, productivity loss due to presenteeism; SD, standard deviation; OR, odds ratio; CI, confidence interval

Similarly, in the sensitivity analysis, no significant difference was observed in the monotherapy, but the odds of presenteeism loss were significantly higher in combination therapy, especially in the intermediate control group and in the poor control group (Supplemental Table 2).

## Discussion

In our previous study [16], the diabetic drug treatment group had a significantly higher presenteeism loss compared with that of the normal group, and therefore we focused on two severities, “treatment control status” and “number of anti-diabetic drugs.” Here we investigated the relationship between these severities and presenteeism loss for type 2 diabetes mellitus.

Our results of the severity in the diabetic treatment group suggested that presenteeism loss was less likely to occur with monotherapy, but combination therapy could cause the loss even when treatment control was good, and further loss could occur when treatment control became poor, specifically HbA1c ≥ 7%.

### Occurrence of presenteeism loss for severity of diabetes

There are several possible factors that cause presenteeism loss due to the severity of diabetes. Poor treatment control tends to cause typical diabetic symptoms such as dry mouth and polydipsia [38], and these symptoms may cause presenteeism. In addition, poor treatment control and longer treatment periods increase the risk of developing diabetes-related complications such as heart disease, cerebrovascular disease, neuropathy, nephropathy, retinopathy and diabetic foot ulces [26, 32, 39–42].

It can be difficult to control treatment over a long time period, and therefore multiple prescriptions may be required [32]. Although we did not identify symptoms or complications in this study, these could be present in the case of intermediate and poor treatment control, or when the treatment period has been long and multiple prescriptions have been required for treatment control.

Diabetes treatment often causes diabetes-associated stress, including stress due to poor glycemic control, and concerns about the need to continue treatment, future complications and costs [43]. In particular, the psychological burden of the poor control group may be large [44, 45]. Diabetes is often associated with mental health challenges such as depression and anxiety [46, 47]. Presenteeism loss is likely to occur due to the diabetes-associated stress and the complications of the mental difficulties [28, 29, 30], which may be due to the severity of diabetes treatment causing presenteeism through psychiatric symptoms.

If poor control continues, more anti-diabetic drugs may be used in an attempt to improve control or dosage might be increased. In this study, this was true because the proportion of combination therapy was higher in the poor control group. The previous study reported that as the number of tolerability issues associated with anti-diabetic drugs such as hypoglycemia, gastrointestinal symptoms, weight gain, edema, etc. increased, presenteeism loss was more likely to occur [31], and combination therapy may increase these issues compared with monotherapy [48]. Furthermore, combination therapy may cause issues that are unlikely to occur with monotherapy. For example, dipeptidyl peptidase IV inhibitors [49, 50] and sodium-glucose cotransporter 2 inhibitors [51], which are currently the mainstream prescriptions in Japan, are unlikely to cause hypoglycemia by themselves, but it is reported that the combined use of sulfonylureas and insulin is more likely to cause hypoglycemia, and the guidelines also draw attention to this likelihood [34].

Thus, there was no significant difference between the monotherapy and normal group and the loss that occurred, although this was higher in the combination therapy group in this study. The presenteeism loss may have occurred through tolerability issues due to the increase in the number and dose of anti-diabetic drugs. Another factor is simply the burden of increasing the number of oral doses and the number of tablets taken at one time.

### Use of the results

As diabetes becomes more severe, in addition to the direct effects of poor glycemic control, the number of anti-diabetic drugs and dosages increase, causing multiple tolerability issues and diabetes-associated stress. This may then lead to a vicious cycle of reduced exercise and diet behavior, medication adherence, and treatment satisfaction, resulting in further poor glycemic control [43, 52, 53]. As our findings suggested that the severity of diabetes might cause presenteeism loss, from the perspective of workplace health and productivity management intervention efforts in the workplace should be considered. Specifically, it is important to detect borderline diabetes early and intervene as soon as possible from this stage. Supporting diet and exercise therapy means that even if type 2 diabetes mellitus is diagnosed, it is not necessary to start an anti-diabetic drug. If an anti-diabetic drug is prescribed, continuous support must be provided to reduce factors that hinder the continuation of treatment and glycemic control so that the treatment can be continued and controlled by monotherapy. Diabetes treatment is linked to psychological burden, and therefore it is also necessary to consider providing regular mental health support [32].

### Strengths and limitations

This study revealed that the severity of diabetes might have a significant effect on the relationship between diabetes and presenteeism. Many previous studies of these relationships selected participants and evaluated treatment status through e-mail and using the Internet. However, in this study, we used objective data based on the results of medical examinations and health insurance claims data.

There were several limitations to this study. First, the duration of diabetes was not taken into consideration. In general, longer durations of the condition mean greater possibility of having diabetic complications such as neurosis, retinopathy, and nephropathy. Second, annual income and education, which were considered as confounding factors, have not been obtained in this study and could not be added to the adjusting factors. However, since the workers of large companies were the target in this study and the majority of them were university graduates or above, the influence of their education was considered to be small. Third, the evaluation index of presenteeism loss using the QQ method is a self-administered survey, which reflects the participants’ subjectivity so that presenteeism loss may be over- or underestimated. In addition, a recall bias may have occurred because we asked the symptoms in the past month. However, there was no difference in the average number of days of symptoms between the treatment control groups, so it was unlikely that it had a significant effect on the results. Forth, this study was a cross-sectional study, which means that recent changes in treatment content have not been captured. Fifth, because this study targeted workers in large companies with very high health literacy, it was considered that the system for follow-up of medical examinations and the environment that makes it easy to visit hospitals were in place, and that employees may have visit a hospital and receive treatment immediately. So, it is difficult to generalize our results to small and medium-sized companies and it is necessary to expand the target participants and carry out further research.

## Conclusion

Our results found that there was an association between the severity of diabetes measured by treatment control status and number of treatments, and occurrence of presenteeism loss. Higher severity was more likely to cause loss among the diabetic drug treatment groups. It was also suggested that even if treatment control was good, the loss could occur when the number of anti-diabetic drugs was high. Therefore, in the workplace, it is important to provide early intervention and continuous support as a preventive measure against diabetes and diabetes-related complications of workers, and also as a measure against presenteeism, which causes large losses for companies.

## Supplementary Information

**Additional file 1.** Supplemental Table 1. Relationship between each treatment control and high presenteeism loss (the top 10% of presenteeism loss)

**Additional file 2.** Supplemental Table 2. Relationship between the number of anti-diabetics and high presenteeism loss (the top 10% of presenteeism loss).

## Data Availability

The datasets used and/or analyzed during the current study are available from the corresponding author on reasonable request.

## Abbreviations

IDF: International Diabetes Federation
HbA1c: hemoglobin A1c
NGSP: National Glycohemoglobin Standardization Program
QQ: Quantity and Quality
SD: standard deviation
OR: odds ratio
CI: confidence interval
BMI: body mass index
