# Supplementary material for: Diabetes severity measured by treatment control status and number of anti-diabetic drugs affects presenteeism among workers with type 2 diabetes
Source: BMC Public Health. 2021 Oct 16;21:1865. doi: 10.1186/s12889-021-11913-3 (PMC8520264; doi:10.1186/s12889-021-11913-3)
Supplement: Supplementary file 1 — Additional file 1. Supplemental Table 1. Relationship between each treatment control and high presenteeism loss (the top 10% of presenteeism loss) [file 12889_2021_11913_MOESM1_ESM.docx]

Supplemental Table 1. Relationship between each treatment control and high presenteeism loss (the top 10% of presenteeism loss)

|  |  |  | n | mean (SD) of  presenteeism loss | % | Crude model | | |  | Adjusted model | | |
| --- | --- | --- | --- | --- | --- | --- | --- | --- | --- | --- | --- | --- |
|  |  |  |  |  |  | OR | 95% CI | *p*-value |  | OR | 95% CI | *p*-value |
| Normal group | | | 11494 | 16.8 (24.4) | 13.5 | reference |  |  |  | reference |  |  |
| Good control group | | | 300 | 17.6 (24.8) | 13.7 | 1.01 | 0.73–1.42 | 0.213 |  | 1.34 | 0.94–1.92 | 0.110 |
| Intermediate control group | | | 105 | 21.8 (27.7) | 18.1 | 1.42 | 0.86–2.33 | 0.173 |  | 1.63 | 0.95–2.82 | 0.079 |
| Poor control group | | | 80 | 22.7 (30.7) | 21.3 | 1.73 | 1.01–2.96 | 0.046 |  | 1.81 | 0.98–3.33 | 0.058 |
| Adjusted model controlled for sex, age, employment status, occupation and company.  Presenteeism loss, productivity loss due to presenteeism; SD, standard deviation; OR, odds ratio; CI, confidence interval | | | | | | | | | | | | |
