# Supplementary material for: Diabetes severity measured by treatment control status and number of anti-diabetic drugs affects presenteeism among workers with type 2 diabetes
Source: BMC Public Health. 2021 Oct 16;21:1865. doi: 10.1186/s12889-021-11913-3 (PMC8520264; doi:10.1186/s12889-021-11913-3)
Supplement: Supplementary file 2 — Additional file 2. Supplemental Table 2. Relationship between the number of anti-diabetics and high presenteeism loss (the top 10% of presenteeism loss). [file 12889_2021_11913_MOESM2_ESM.docx]

Supplemental Table 2. Relationship between the number of anti-diabetics and high presenteeism loss (the top 10% of presenteeism loss)

|  |  |  | n | mean (SD) of  presenteeism loss | % | Crude model | | |  | Adjusted model | | |
| --- | --- | --- | --- | --- | --- | --- | --- | --- | --- | --- | --- | --- |
|  |  |  |  |  |  | OR | 95% CI | *p*-value |  | OR | 95% CI | *p*-value |
| Normal group | | | 11494 | 16.8 (24.4) | 13.5 | reference |  |  |  | reference |  |  |
| Monotherapy group | | | 190 | 17.1 (24.1) | 13.7 | 1.02 | 0.67–1.54 | 0.942 |  | 1.14 | 0.72–1.82 | 0.571 |
| Combination therapy group | | | 295 | 20.8 (27.9) | 17.3 | 1.34 | 0.99–1.82 | 0.062 |  | 1.71 | 1.23–2.39 | 0.002 |
|  | Good control group | | 154 | 18.3 (25.6) | 13.6 | 1.01 | 0.64–1.61 | 0.962 |  | 1.46 | 0.90–2.37 | 0.127 |
|  | Intermediate control group | | 74 | 23.9 (29.0) | 21.6 | 1.77 | 1.01–3.08 | 0.045 |  | 1.98 | 1.07–3.67 | 0.029 |
|  | Poor control group | | 67 | 23.3 (31.3) | 20.9 | 1.69 | 0.94–3.06 | 0.081 |  | 1.96 | 1.03–3.72 | 0.039 |
| Adjusted model controlled for sex, age, employment status, occupation and company.  Presenteeism loss, productivity loss due to presenteeism; SD, standard deviation; OR, odds ratio; CI, confidence interval | | | | | | | | | | | | |
